# Frailty Severity Outcomes After Left Atrial Appendage Occlusion

**DOI:** 10.1016/j.jacadv.2026.102963

**Published:** 2026-06-26

**Authors:** Osamah Badwan, Chadi Tabaja, Issam Motairek, Abdallah Abuawad, Faysal Massad, Grant Reed, Abdulla Damluji, Mohamed Kanj, Oussama Wazni, Samir Kapadia

**Affiliations:** Cardiovascular Center on Aging, Department of Cardiovascular Medicine, Heart, Vascular and Thoracic Institute, Cleveland Clinic, Cleveland, Ohio, USA

**Keywords:** biological age, cardiovascular outcomes, frailty, geriatric syndromes, left atrial appendage occlusion, modified frailty index

**What is the clinical question being addressed?**

Does frailty severity predict long-term clinical and geriatric outcomes after left atrial appendage occlusion?

**What is the main finding?**

Increasing frailty severity was associated with stepwise higher mortality, ischemic stroke, cardiovascular events, and geriatric syndromes, supporting frailty assessment before left atrial appendage occlusion.

Left atrial appendage occlusion (LAAO) is an established strategy for stroke prevention in patients with atrial fibrillation (AF) who are unsuitable for long-term anticoagulation. However, patients undergoing LAAO in contemporary practice are often older and medically complex, and chronological age alone may not adequately capture procedural vulnerability. Frailty is a multidimensional marker of diminished physiologic reserve and has shown prognostic value across cardiovascular interventions.^1^ We therefore evaluated whether frailty severity, measured by the 5-item modified frailty index (MFI-5), can predict long-term clinical and geriatric outcomes after LAAO.

We performed a retrospective cohort study using the TriNetX Research Network. Adults with nonvalvular AF undergoing percutaneous LAAO between 2015 and 2024 were identified using procedural codes. Patients with rheumatic mitral stenosis or mechanical prosthetic heart valves were excluded. Frailty was assessed using the MFI-5, which includes hypertension, diabetes mellitus, chronic obstructive pulmonary disease, congestive heart failure, and nonindependent functional status. Patients were categorized as robust (MFI-0), prefrail (MFI-2), and frail (MFI-5). To approximate an intermediate frailty phenotype (prefrail), patients were identified using combinations of MFI-5 components and the total number of MFI-related comorbidities across the cohort was divided by the number of patients, yielding an average frailty burden of approximately 2 components per patient. Propensity score matching was performed separately for each pairwise comparison using 1:1 nearest-neighbor matching without replacement and a caliper width of 0.1 pooled SDs. Covariates included age, sex, race, chronic kidney disease, heart failure, atherosclerotic heart disease, prior stroke, tobacco use, body mass index, and estimated glomerular filtration rate. Balance between matched cohorts was assessed using standardized mean differences, with values <0.10 considered indicative of acceptable covariate balance. Because the analysis was conducted using deidentified data, Institutional Board Approval was waived.

After propensity matching, cohorts were generally balanced across age, sex, race, and major cardiovascular comorbidities. The mean age ranged from 74 to 77 years; women comprised 42% to 46% and the majority were White.

Clinical and geriatric outcomes across frailty strata are summarized in Figure 1. Increasing frailty severity was associated with a stepwise increase in adverse outcomes. Compared with robust patients (MFI-0), prefrail patients (MFI-2) had higher all-cause mortality (11.3% vs 4.5%; HR: 1.96; 95% CI: 1.45-2.63), whereas frail patients (MFI-5) experienced substantially higher mortality (35.2% vs 4.1%; HR: 9.09; 95% CI: 5.56-14.29). When frail and prefrail groups were compared directly, frail patients remained at significantly higher risk of death (33.6% vs 13.5%; HR: 3.03; 95% CI: 2.38-3.70).

**Figure 1:**
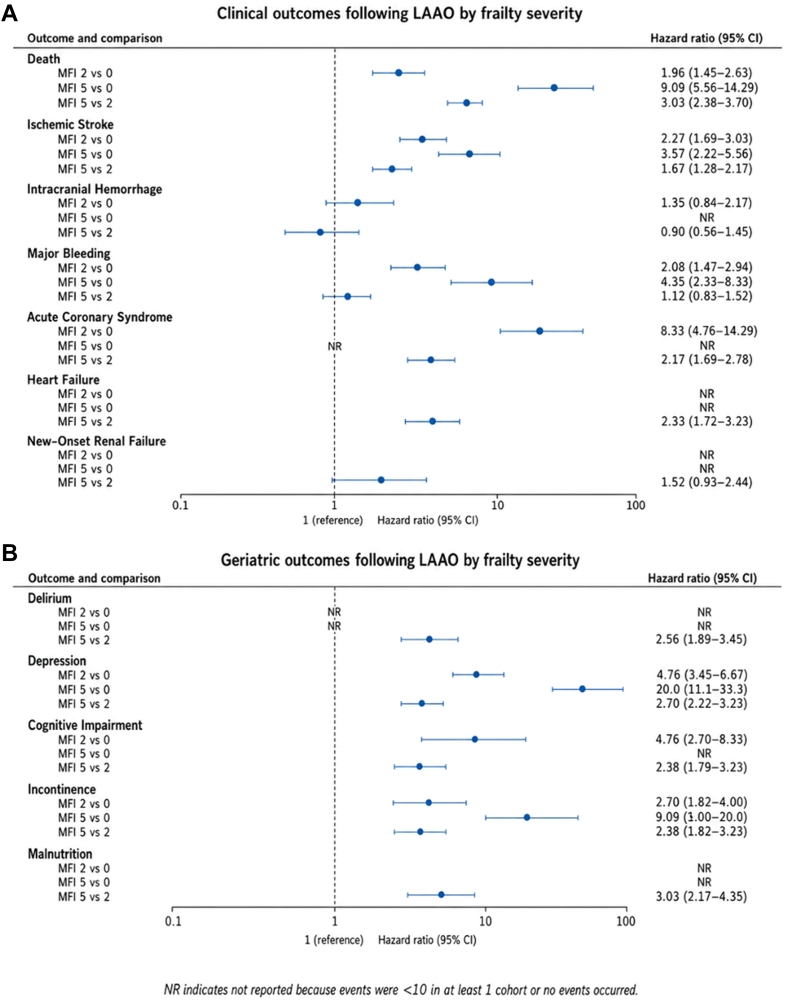
Frailty Severity and Outcomes After Left Atrial Appendage Occlusion Forest plots show HRs and 95% CIs for propensity score-matched pairwise comparisons among robust (MFI-0), pre-frail (MFI-2), and frail (MFI-5) cohorts. Panel A summarizes clinical outcomes and B summarizes geriatric outcomes. Effect estimates are reported such that higher frailty categories are compared against lower frailty categories. Outcomes with fewer than 10 events per cohort were not reported. LAAO = left atrial appendage occlusion; MFI = modified frailty index.

A similar pattern was observed for ischemic stroke. Compared with robust patients, prefrail patients had a higher risk of stroke (11.3% vs 4.4%; HR: 2.27; 95% CI: 1.69-3.03), and this risk was further accentuated among frail patients (17.5% vs 4.9%; HR: 3.57; 95% CI: 2.22-5.56). Stroke risk also remained higher among frail patients compared with prefrail patients (18.0% vs 12.0%; HR: 1.67; 95% CI: 1.28-2.17). Prefrail and frail patients also demonstrated higher major bleeding risk relative to robust patients, whereas bleeding rates were comparable between frail and prefrail groups. Frail patients further exhibited higher risks of acute coronary syndrome and heart failure hospitalization compared with prefrail patients, whereas new-onset renal failure was numerically more frequent but did not reach statistical significance. Intracranial hemorrhage rates were low and similar across frailty groups.

Higher frailty severity was likewise associated with greater geriatric syndrome burden. Compared with prefrail patients, frail patients had significantly higher risks of delirium, depression, cognitive impairment, incontinence, and malnutrition. Compared with robust patients, both prefrail and frail patients had markedly higher risks of depression and incontinence.

In prespecified age-stratified analyses, the association between frailty and adverse outcomes persisted and appeared most pronounced among patients >75 years of age. However, these findings should be considered exploratory because formal interaction testing was not available within the TriNetX platform. In the MFI-0 vs MFI-5 comparison, several secondary endpoints could not be estimated because event counts were below the TriNetX minimum reporting threshold of 10.

In this large real-world cohort of patients undergoing percutaneous LAAO, increasing frailty burden as quantified by the MFI-5 was strongly associated with adverse long-term clinical and geriatric outcomes. Importantly, these associations were evident not only among frail patients, but also in those with intermediate frailty, underscoring that frailty exists along a continuum rather than as a binary state.

To our knowledge, this is the first real-world study to evaluate graded frailty severity and its association with long-term clinical and geriatric outcomes following LAAO. These findings extend prior work showing that frailty improves prognostic discrimination in structural heart interventions.^2^^,^^3^ Although frailty has been well studied in transcatheter aortic valve replacement populations, data in LAAO remain limited.

Our results suggest that the prognostic relevance of the MFI-5 is consistent across structural heart procedures despite differences in procedural complexity and patient selection. Notably, most patients undergoing LAAO in contemporary practice appeared to fall within the prefrail category (MFI-2 comprised 95% of the overall cohort), whereas the frail cohort represented only a small proportion of the overall population, reflecting that current LAAO practice predominantly involves patients with intermediate comorbidity burden. Nevertheless, findings from the MFI-0 vs MFI-5 comparison should be interpreted cautiously, as this matched cohort was the smallest and several secondary endpoints were not estimable because of low event counts.

Beyond conventional cardiovascular endpoints, frailty was associated with a markedly higher burden of geriatric syndromes, including delirium, depression, cognitive impairment, incontinence, and malnutrition. These outcomes are rarely emphasized in LAAO studies yet have major implications for quality of life, functional independence, and health care use. In this context, the MFI-5 offers a pragmatic and scalable approach to risk stratification in a real-world population, even if it does not capture performance-based or social domains of frailty.

These findings also raise important ethical considerations regarding patient selection and potential procedural futility in highly frail individuals undergoing LAAO. Although LAAO is an effective strategy for stroke prevention in patients with AF who are unsuitable for long-term anticoagulation, the substantially higher mortality observed among frail patients suggests that the long-term benefits of stroke prevention may be attenuated by competing risks of death and overall comorbidity burden. In such circumstances, the balance between procedural benefit and overall prognosis becomes particularly relevant. Incorporating frailty assessment into the preprocedural evaluation may therefore facilitate more individualized risk-benefit discussions and shared decision-making with patients and families regarding the expected benefits of LAAO.

This study has several important limitations. First, frailty was assessed using the MFI-5, a pragmatic comorbidity-based instrument that does not capture performance-based, cognitive, or social domains of frailty. Second, this analysis relied on aggregate electronic health record data from the TriNetX Research Network and is therefore subject to coding variability, underreporting, and limited clinical granularity. Third, although patients in the prefrail group were intended to represent an intermediate frailty phenotype, the specific combinations of MFI-5 components were heterogeneous and may not carry equivalent prognostic weight. Fourth, because the cohort was restricted to patients undergoing LAAO, the study is subject to inherent procedural selection bias and does not permit comparison with medically managed AF populations. For the MFI-0 vs MFI-5 comparison, low event counts reflect the small matched cohort (N = 469) and paradoxically low postprocedural event rates in the highly selected robust comparator after matching across highly dissimilar populations, a recognized limitation of propensity matching. As with all electronic health record–derived observational studies, unmeasured confounding remains possible despite matching, including factors such as socioeconomic status, medication adherence, procedural selection patterns, and frailty dimensions not captured through administrative coding.

Finally, frailty was analyzed categorically; sensitivity analyses with continuous or ordinal MFI and Fine-Gray competing risk models were not feasible in TriNetX; the proportional hazards assumption could not be formally tested; age-stratified analyses were exploratory without formal interaction testing; and unmeasured confounding from procedural selection, unrecorded functional determinants, and differential ascertainment cannot be excluded.

In this large real-world cohort of patients undergoing LAAO, frailty severity was a powerful predictor of long-term clinical and geriatric outcomes. Prefrail patients comprised approximately 95% of the LAAO cohort, confirming the MFI identifies clinically meaningful gradations of vulnerability within an already-comorbid population. These findings support incorporating frailty assessment into preprocedural evaluation to improve risk stratification, patient selection, and shared decision-making.

## Funding support and author disclosures

This work was supported by unrestricted philanthropic support to the Cleveland Clinic Heart, Vascular, and Thoracic Institute. The funding source had no role in the design or conduct of the study; the collection, management, analyses, or interpretation of the data; the preparation, review, or approval of the manuscript; or the decision to submit the manuscript for publication. The authors have reported that they have no relationships relevant to the contents of this paper to disclose.
